# Antifungal Susceptibility Patterns of Vulvovaginal *Candida* species among Women Attending Antenatal Clinic at Mbarara Regional Referral Hospital, South Western Uganda

**DOI:** 10.9734/BMRJ/2015/13804

**Published:** 2014-11-06

**Authors:** Kiguli James Mukasa, Itabangi Herbert, Atwine Daniel, Kibuka Livingstone Sserunkuma, Bazira Joel, Byarugaba Frederick

**Affiliations:** 1 Department of Microbiology, Mbarara University of Science and Technology, Mbarara Uganda; 2 Department of Clinical Research, Epicentre Mbarara Research Centre, Mbarara, Uganda; 3 Department of Community Health, Mbarara University of Science and Technology, Mbarara, Uganda; 4 Department of Microbiology, Makerere College of Health Sciences, Makerere University, Kampala, Uganda

**Keywords:** Antifungal, susceptible, resistant, vulvovaginal candidiasis, Candida species

## Abstract

**Aims:**

To identify the *Candida* species that cause vulvovaginal candidiasis and determine their antifungal susceptibility patterns.

**Study Design:**

This was a cross-sectional study.

**Place and Duration of Study:**

The study was conducted at the antenatal clinic of Mbarara Regional Referral Hospital in Mbarara Municipality, between December 2012 and February 2013.

**Methods:**

High vaginal swabs from 456 pregnant women were subjected to microscopy and culture on Sabouraud Dextrose Agar. *Candida* isolates were identified by the germ tube and Analytical profile index (API^®^ Candida) tests. Susceptibility to fluconazole, itraconazole and voriconazole was determined by the Etest strips and for clotrimazole and nystatin by the disc diffusion method on Mueller Hinton agar supplemented with 2%w/v glucose and 0.5μg/ml methylene blue dye.

**Results:**

Of the 456 High vaginal swabs cultured, 207 grew *Candida species.* Species distribution was as follows: *C. albicans* (78.95%), *C. glabrata* (14.35%), *C. krusei* (3.35%), *C. tropicalis* (1.44%), *C. famata* (0.96%), *C. parapsilosis* (0.48%) and *C. lusitaniae* (0.48%). Resistance to nystatin was only observed in 0.61% of *C.albicans*. Resistance to clotrimazole was observed in 50%, 36.67% and 0.61% of *C. famata*, *C. glabrata* and *C. albicans* respectively. *C. krusei* showed a high resistance of 71.43% to fluconazole. *C. glabrata*, *C. krusei*, *C. famata* and *C. lusitaniae* exhibited 100% resistance to itraconazole. Resistance to voriconazole of less than 11% was exhibited by only *C. albicans* and *C. glabrata.*

**Conclusion:**

*C.albicans* was susceptible to most antifungal agents tested except itraconazole and voriconazole. All isolates were susceptible to nystatin except less than 1% of *Candida albicans*. Non-*albicans* demonstrated resistance to some drugs especially itraconazole. We recommend use of Nystatin for empirical management of vulvovaginal candidiasis among pregnant women.

## 1. INTRODUCTION

Vulvovaginal candidiasis (VVC) is an opportunistic fungal infection of the female lower genital tract caused by *Candida* species [1]. Mucosal candidiasis, especially vulvovaginal candidiasis, is the most common fungal disease in normal healthy women [2,3] On the other hand pregnant women are the most predisposed to severe vulvovaginal candidiasis as a result of lowered immunity [4–6] and this severity may involve among others chorioamnionitis which eventually leads to preterm labour [7,8]. According to available data, preterm birth is the leading cause of neonatal mortality worldwide [9]. *Candida albicans* is a dimorph and an endogenous commensal implicated as an etiologic agent in most cases of the female lower reproductive tract infections [2,10]. Other non-*albicans* species such as *C. glabrata, C. parapsilosis and C. krusei* although rare compared to *C. albicans,* may also cause serious opportunistic infections and exhibit more resistance to a number of antifungal agents.

Although there are many effective antifungal drugs both topical and oral that are used for treating candidal vulvovaginitis, reduced susceptibility of some vaginal yeasts isolates to some antifungal agents has been reported [11] in Costa Rica involving yeasts isolated from high vaginal swabs.

Understanding of the antifungal susceptibility patterns of pathogenic fungi is key in guiding appropriate therapy selection for mycoses [12–15]. Antifungal susceptibility testing may also provide an estimation of antifungal effectiveness, ensuring good treatment outcome, monitoring of development of drug resistance and therapeutic potential of unproven compounds [12–14].

In Uganda data on the species and susceptibility of vulvovaginal yeasts to antifungal drugs is limited. Routine species typing and in vitro antifungal susceptibility testing of *Candida* isolates prior to therapy has not been given its appropriate attention to determine the occurrence of resistant genotypes and consequently, there is no data about prevalent *Candida* species and their antifungal susceptibility patterns in this region. Therefore, the aim of this study was to perform speciation of *Candida* isolates responsible for causing VVC and determine their susceptibility patterns to the commonly used antifungal drugs in Mbarara, Uganda.

## 2. MATERIALS AND METHODS

### 2.1 Study Area

The study was conducted at the antenatal clinic (ANC) of Mbarara Regional Referral Hospital in Mbarara Municipality. Mbarara Regional Referral Hospital is also a teaching Hospital for the Medical School of Mbarara University of Science and Technology. At the antenatal clinic the daily attendance ranges between 40 and 60 pregnant women.

The High vaginal swabs (HVS) were processed from the Microbiology Laboratory at Mbarara University of Science and Technology where clinical specimens from the Hospital are processed.

#### 2.1.1 Study population

The study subjects were pregnant women who had presented to the Mbarara Regional Referral Hospital antenatal clinic for ante-natal care.

#### 2.1.2 Inclusion criteria

The consented pregnant women of age 18years and above who presented at the antenatal clinic for antenatal care were included in the study. Those aged below 18years were also included and considered emancipated minors.

#### 2.1.3 Exclusion criteria

Pregnant women who did not consent and those who reported a history of vaginal bleeding were excluded from the study.

#### 2.1.4 Sampling procedure

Pregnant women at the ANC were selected and recruited into the study by convenience sampling after a health talk about the study had been given by the principal investigator or the nurse on duty.

#### 2.1.5 Consent and counseling

A written consent was sought from those pregnant women who satisfied the inclusion criteria and this was followed by filling in the questionnaire, and counseling for specimen collection. The study subject was then sent to a private room for specimen collection. The results were kept confidential under key and lock.

#### 2.1.6 Questionnaire

This tool was used to capture demographic data and predicting factors for vulvovaginal candidiasis.

#### 2.1.7 Collection of high vaginal swabs

Two High vaginal swabs were taken one after the other by inserting a sterile vaginal speculum into the vagina, and then a sterile cotton wool swab (Copan Italia S.p.A Italy) was inserted into the posterior vaginal fornix and rotated gently before withdrawing. The swab was inserted back into the tube from which it was taken. The tube containing the swab was labelled with the patients study number, initials and date.

### 2.2 Examination of High Vaginal Swabs

One swab was examined by wet preparation method and Gram stain to give the preliminary results to the clinician for patient management. A few drops of sterile normal saline were added to one of the swabs in the tube and shaken to dislodge materials from the swab into the saline. The swab was withdrawn and a sterile inoculating loop was used to transfer and spread a small amount of the deposit onto a clean dry slide to make a smear. The smear was allowed to air-dry, stained by Gram’s method and then examined under the microscope for yeast cells using the oil immersion objective lens.

A wet film was prepared by placing a drop of the saline deposit from the tube onto a clean dry glass slide then covered with a cover slip and examined under the microscope for budding yeast cells and pseudohyphae first using the 10X objective before switching to 40X objective lens to confirm the presence or absence of yeasts.

#### 2.2.1 Culture and isolation of yeast cells

The second swab was streaked onto a plate of Sabouraud dextrose agar (SDA) with chloramphenicol (Biolab Hungary) and incubated at 37°C for 48 hours. Pure yeast colonies were isolated and a Gram stain done to confirm they were yeast cells.

### 2.3 Preservation of Yeast Cells

By use of a sterile wire loop, the yeast colonies were scraped from the SDA plate and suspended in1ml of 16.8%v/v glycerol then stored in a −80°C deep freezer till needed for identification and susceptibility testing.

### 2.4 Subculture and Species Identification

Subculture of preserved yeasts was done on SDA free from chloramphenicol (Mast Group Ltd. Merseyside, UK.) medium, and inoculated plates were incubated at 37°C for 18 to 24 hours. Identification was then carried out by the germ tube test and API analysis.

#### 2.4.1 Germ tube test

The germ tube test was performed by dispensing 0.5ml of freshly prepared human serum into sterile 1.25ml cryovial. Using a sterile wire loop the serum was inoculated with a yeast colony from the SDA plate and the cover loosely placed to allow an aerobic condition. The cryovial was placed in an incubator at 37°C for 3 hours. Using a Pasteur pipette, a drop of the serum yeast culture was transferred to a glass slide, and covered with a cover glass.

The preparation was examined using the ×10 and ×40 objectives with the condenser diaphragm closed sufficiently to give a good contrast. A search was done for sprouting yeast cells, which were tube like outgrowths from the cells (known as germ tubes).

If the sprouting cells were seen the culture was reported as ‘*C. albicans* isolated’ and if no sprouting seen reported as ‘yeast other than *C. albicans* isolated’.

#### 2.4.2 Analytical Profile Index test strip method

All the yeast isolates were identified to species level by Analytical Profile Index strips (API^®^ *Candida* BioMerieux^®^ SA) according to the manufacturer’s procedure.

An incubation tray was prepared by adding 5mls of distilled water into the honey-combed wells of the tray to create a humid atmosphere then the isolate identification number was recorded on the elongated flap of the tray. An API strip was removed from its individual packaging and put into the tray. A few Yeast colonies of an18 to 24 hour culture were picked from SDA plate using a sterile cotton swab and emulsified into 2mls of sterile 0.85%w/v sodium chloride and agitated using a vortex mixer to disperse the cells. The turbidity of the suspension was measured using a densimat (BioMerieux) and adjusted to a 3.0 McFarland number using sterile 0.85%w/v sodium chloride, and used immediately.

The yeast suspension was mixed well, and dispensed into the tubes of the API strip using a 1000μl pipettor with sterile tips care being taken to avoid air bubbles. The first five tubes of glucose to raffinose and the last tube of urea were covered with a layer of mineral oil immediately after inoculating the strip.

The cover of the tray was put in place and the strip incubated at 37°C in an aerobic condition for 18 – 24 hours.

#### 2.4.3 Reading and Interpretation of the strip

After 18 – 24 hours of incubation the reaction in the strip was read referring to the interpretation table in the package insert and recorded as + or − on the result sheet.

The reactions were coded into a numerical profile on the result sheet. The tests were separated into groups of three and a number 1, 2 or 4 assigned to each one. By adding together the numbers corresponding to positive reactions within each group, a 4-digit numerical profile was obtained.

Identification was performed by looking up the profile in the list of the package insert, and also with the apiweb^™^ identification software by entering the 4-digit profile manually via the key board into the mini-API analyzer (BioMerieux) that gave a print out of the species identification.

### 2.5 Antifungal Susceptibility Testing

Antifungal susceptibility testing was done by agar diffusion method. Etest strips (BioMerieux SA) for fluconazole (0.016–256 μg/ml), itraconazole (0.002–32 μg/ml) and voriconazole (0.002–32 μg/ml) were used. For clotrimazole (50μg) and nystatin (100 IU) antifungal discs (Bio-Rad Laboratories Pvt Ltd. France) were used. Mueller-Hinton Agar (Mast Group Ltd., Merseyside UK.) supplemented with 2%w/v glucose and 0.5μg/ml methylene blue dye was used as recommended by CLSI document M44A [16].

#### 2.5.1 Epsilometer and Disc diffusion method

A yeast colony of 18 to 24 hour incubation culture was picked from the SDA plate using a sterile cotton swab and emulsified into 2mls of 0.85%w/v sterile sodium chloride and agitated on the vortex mixer to achieve a homogeneous suspension.

The turbidity of the suspension was measured using a densimat (BioMerieux) and adjusted to a 0.5 McFarland number using sterile saline.

A sterile cotton swab was moistened in the adjusted inoculum suspension then excess moisture was removed by rolling the swab on the inside of the tube above fluid level.

The surface of Mueller Hinton-glucose agar plate was streaked in 4 different directions (at 90 degree angles) to cover the entire surface. The plate was left to dry at 35°C until no droplets of moisture were on the agar surface. Using a pair of flame sterilized forceps an Etest strip or disc of the antifungal agent was applied onto the surface of the inoculated agar plate and pressed lightly to ensure complete contact with agar.

The plates were incubated at 35°C for 24 to 48 hours or until sufficient growth had occurred. Plates were read as early as possible after 24 hours incubation and results recorded.

**Fig. 1:**
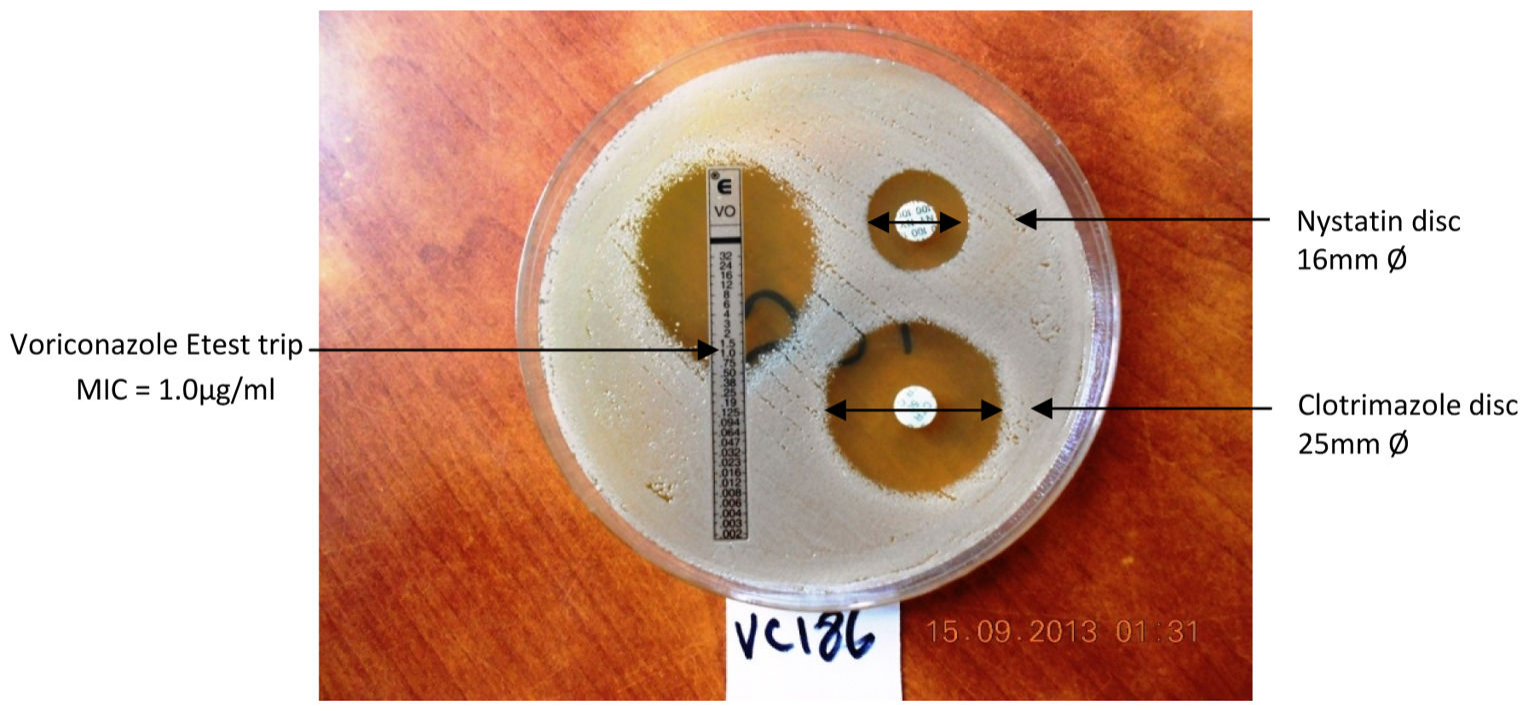
Zones of inhibition

The Minimum Inhibitory Concentration (MIC) value was read at the point where the inhibition ellipse intersected the strip (Fig. 1). Etest strips results were interpreted according to the manufacturer’s interpretation criteria as susceptible, susceptible dose dependent (S-DD) or resistant with MIC values as indicated in Table 1. For antifungal discs the diameters of zones of inhibition were measured in millimeters using a ruler. The results were interpreted according to the manufacturer’s interpretation criteria as sensitive, intermediate or resistant as indicated in Table 2 below.

### 2.6 Data Analysis

Data was entered into excel and imported into STATA 11.0 for analysis. The baseline characteristics of participants were summarized using the mean, median or proportion as appropriate. Proportions were generated to demonstrate the distribution of *Candida* species. The susceptibility patterns were generated using proportions for each drug type per *Candida* species isolated.

### 2.7 Quality Control/Assurance

New Zealand Reference Culture Collection Medical Section (NZRM) control strains were used. NZRM 3394 (ATCC 90028) *Candida albicans* and NZRM 4072^T^ (ATCC 22019) *Candida parapsilosis* were cultured along with the clinical *Candida* isolates. These strains were used for the validation of identification and susceptibility performance tests. Epsilometer strips were stored between −18°C and −20°C and used as per the manufacturer’s recommendation. The Clotrimazole and Nystatin antifungal discs were stored between +2°C and +8°C.

## 3. RESULTS

### 3.1 Identity and Proportions of *Candida* species

**Fig. 2:**
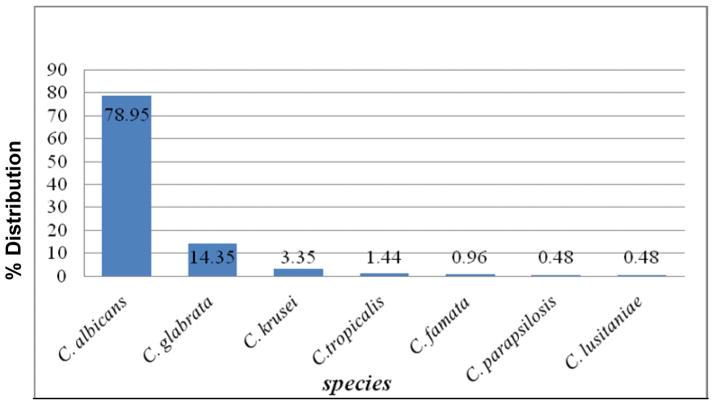
Distribution of *Candida* species

Of the 456 High vaginal swabs cultured 207 grew *Candida* giving a prevalence of 45.4%. Two high virginal swabs yielded two different species of *Candida* giving a total of 209 *Candida* species. *C. albicans* 165 (78.95%) was predominant followed by *C. glabrata* 30 (14.35%), *C. krusei* 7 (3.35%), *C. tropicalis* 3 (1.44%), *C. famata* 2 (0.96%), *C. parapsilosis* 1(0.48%) and *C. lusitaniae* 1 (0.48%) as shown in Fig. 2 below. The overall rate of non albicans isolates was 21.05%.

### 3.2 Antifungal Susceptibility Patterns of Isolated *Candida* species

Antifungal susceptibility testing was done using fluconazole, itraconazole, voriconazole, nystatin and clotrimazole.

**Fig. 3:**
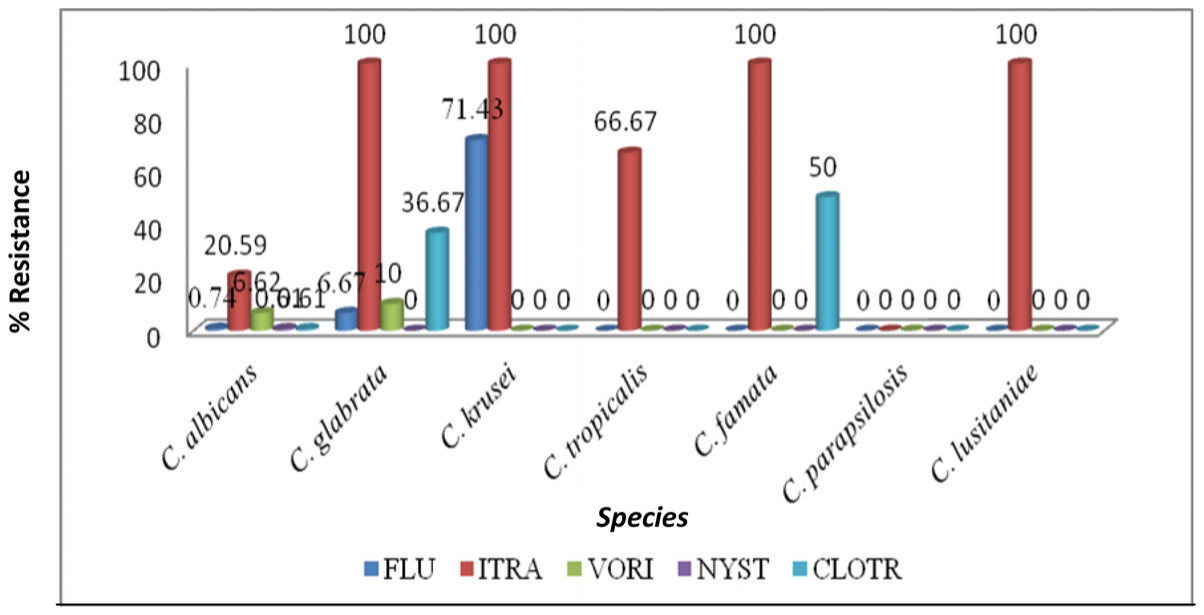
Percentage antifungal resistance of *Candida* species

*C. krusei* showed a high resistance of 71.43% to fluconazole. *C. glabrata*, *C. krusei*, *C. famata* and *C. lusitaniae* exhibited 100% resistance to itraconazole. Resistance to voriconazole and nystatin was minimal. Resistance of all species to nystatin was below 1%. *C. glabrata* and *C. famata* showed a 36.67% and 50% resistance to clotrimazole respectively. *C. albicans* exhibited resistance of 20.59% to itraconazole and 6.62% to voriconazole as shown in Fig. 3 below.

## 4. DISCUSSION

### 4.1 Species Identification and Distribution

Vulvovaginal candidiasis is a common female genital problem that affects women especially of child bearing age and pregnant women [4]. Pregnant women are more susceptible to both vaginal colonization and infection by yeasts as a result of high concentration of oestrogen hormone during pregnancy which provides a suitable environment for the growth of *Candida* [17]. In this study the center of attention was pregnant women with the broad objective to identify the *Candida* species that cause vulvovaginal candidiasis and determine their antifungal susceptibility patterns.

Data on the prevalence of Ugandan *Candida* species causing vulvovaginitis remains scarce since cultures are rarely performed. Our study showed that *C. albicans* was the most prevalent species (79%). The overall prevalence of non-albicans species was 21% with *C. glabrata* the most predominant. This dominancy agrees well with findings of 20% from another study [18], but differs from some other two studies [19,20] who found lower values of 17% and 11% respectively

The findings in our study are similar to those of the study in Kenya [21] which showed that of the *Candida* species isolated, *C.albicans* were the most common (73.7%). Among the non *albicans* species, *C. glabrata* (13%), *C. famata* (5%), *C. krusei* (3%) and *C. parapsilosis* (1%) were the species isolated.

Furthermore, our study findings are also similar to those of the study in Brazil [22] about species distribution from pregnant women, where, *C. albicans* were 83 (92.3%), *C. krusei* 3 (3.3%), *C. glabrata* 2 (2.2%), *C. parapsilosis* 1 (1.1%), and 1 (1.1%) as *C.tropicalis*. When we look at this species distribution in our study and other similar studies, it is worth noting that *C. albicans* is the most prevalent compared with other species, the reason probably being that it is a normal flora that takes advantage of risk factors such as pregnancy, antibiotic therapy, uncontrolled diabetes mellitus, immunosupression due to HIV and others.

Other than *C. albicans*, species such as *Candida glabrata* and *Candida tropicalis* are less commonly implicated etiologic agents of vaginal infections and have a propensity to be more resistant to treatment. On the other hand, our study findings differ from those of a recent study in India [23] where only five *Candida* species were isolated and identified. Their study results further showed a lower proportion of *Candida albicans* (35.5%), followed by higher incidence of non-*albicans* species of *C. tropicalis* (26.4%), *C. glabrata* (20.6%), *C. krusei* (15.7%), and *C. dubliniensis* (1.6%).

This difference shows an increase in frequency of non-*albicans* species particularly, *C. glabrata*, *C. krusei*, and *C. tropicalis* as potential causes of vulvovaginal candidiasis. We contemplate this increasing detection of non *albicans* species is probably related to the widespread and inappropriate use of antimycotic drugs most especially obtained over the counter.

### 4.2 Antifungal Susceptibility Patterns

Antifungal susceptibility testing in our study revealed that most of the *Candida albicans* isolates tested were susceptible to fluconazole, voriconazole, nystatin and clotrimazole. All isolates were susceptible to nystatin with only an exception of less than 1% of *C. albicans*. This finding is in agreement with a similar study carried out in Argentina [17].

In this study the resistance rate of *Candida albicans* to itraconazole was slightly low whereas the susceptible dose dependent was high. In relation to fluconazole, a similar study [24] *C. albicans* showed a very high susceptibility (96%) to fluconazole and MIC values equal to or higher than 64μg/ml were also detected. These percentages are similar to those obtained in our study. As expected, higher azole MICs were found among non *albicans Candida* species. A larger proportion of the *C. glabrata* isolates in this study was not susceptible to fluconazole, itraconazole and clotrimazole and showed the highest susceptible dose dependent rate to fluconazole and absolute resistance rate to itraconazole, followed by clotrimazole resistance and intermediate rate of 60% to the later drug. Similarly, a larger proportion of *C. krusei* was not susceptible to fluconazole and itraconazole and showed very high resistance rates to fluconazole and itraconazole but this is not a surprise because *C. krusei* is intrinsically recognized to be resistant to azoles. *C. tropicalis* isolates showed high resistance to itraconazole and intermediate to clotrimazole. *C. famata* and *C. lusitaniae* showed absolute resistance to itraconazole. *C. famata* showed a 50% intermediate and resistance to clotrimazole. This resistance to itraconazole was high probably because the isolates were few and therefore it may not be worth concluding generally that these species are absolutely resistant to itraconazole. *C. parapsilosis* was susceptible to all other drugs except with an absolute susceptible dose dependent to itraconazole indicating that itraconazole may not be appropriate for the treatment of *C. parapsilosis* infection. These in-vitro results from this study therefore support the use of alternative antimycotic agents when treating vulvovaginal candidiasis caused by non *albicans* species especially *C. glabrata* and *C. krusei*. This study had limited resources that led to the use of a few antifungal agents for susceptibility testing.

## 5. CONCLUSION

In this study the pregnant women presenting with vulvovaginal candidiasis were infected with *C. albicans* as the predominant etiologic agent. The commonest non *Candida albicans* species was *C. glabrata*. Almost all *Candida* isolates were susceptible to nystatin. Resistance to clotrimazole was exhibited by *C. famata*, *C. glabrata* and less than one percent of *Candida albicans*.

## 6. RECOMMENDATION

Nystatin, a relatively cheap and readily available antifungal agent is recommended for empirical treatment where laboratory identification and susceptibility tests cannot be done.

## Figures and Tables

**Table 1:** Interpretation criteria MIC μg/ml for the three Etest strips tested (CLSI 2009)

Antifungal MIC μg/ml	Code	Interpretation criteria MIC μg/ml		
		Susceptible S≤	Susceptible dose dependent S-DD	Resistant R≥
Fluconazole (0.016–256)	FL	8	16–32	64
Itraconazole (0.002–32)	IT	0.12	0.25–0.5	1
Voriconazole (0.002–32)	VO	1	2	4

Reference: Etest® Table 1 summary of Etest® performance, interpretive criteria and Quality Control ranges 16029 B- 2011/01

**Table 2:** Interpretation criteria of diameters of zones of inhibition for the discs

Antifungal drug	Diameter of the zone of inhibition (mm)	MIC (μg/ml)	Interpretation
Nystatin (100 IU)	= 10	-	Resistant
	> 10		Sensitive
Clotrimazole (50μg)	= 20	= 1.56	Sensitive
	20 – 10	1.56 – 6.4	Intermediate
	= 10	= 6.4	Resistant

Reference: Bio rad Laboratories Pvt Ltd. 3, boulevard Raymond Poincaré 92430 Marnes-la-Coquette France
